# A genome-wide DNA methylation signature for *SETD1B*-related syndrome

**DOI:** 10.1186/s13148-019-0749-3

**Published:** 2019-11-04

**Authors:** I. M. Krzyzewska, S. M. Maas, P. Henneman, K. v. d. Lip, A. Venema, K. Baranano, A. Chassevent, E. Aref-Eshghi, A. J. van Essen, T. Fukuda, H. Ikeda, M. Jacquemont, H.-G. Kim, A. Labalme, S. M. E. Lewis, G. Lesca, I. Madrigal, S. Mahida, N. Matsumoto, R. Rabionet, E. Rajcan-Separovic, Y. Qiao, B. Sadikovic, H. Saitsu, D. A. Sweetser, M. Alders, M. M. A. M. Mannens

**Affiliations:** 1 Amsterdam UMC, Department of Clinical Genetics, Genome Diagnostics laboratory Amsterdam, Reproduction & Development, University of Amsterdam, Meibergdreef 9, Amsterdam, The Netherlands; 2 Amsterdam UMC, Department of Pediatrics, University of Amsterdam, Meibergdreef 9, Amsterdam, The Netherlands; 3 Kennedy Krieger Institute, Department of Neurogenetics, 801 N. Broadway, Rm 564, Baltimore, MD 21205 USA; 4 Department of Pathology and Laboratory Medicine, Western University, 800 Commissioner’s Road E, London, ON N6A 5W9 Canada; 5 University Medical Centre Groningen, University of Groningen, Department of Medical Genetics, Hanzeplein 1, 9713 GZ Groningen, The Netherlands; 6 Department of Pediatrics, Hamamatsu University School of Medicine, 1-20-1 Handayama, Higashi-ku, Hamamatsu, 431-3192 Japan; 7 National Epilepsy Centre, NHO, Shizuoka Institute of Epilepsy and Neurological Disorders, 886 Urushiyama, Aoi-ku, Shizuoka 420-8688 Japan; 8 Department of medical genetics, CHU La Reunion-Groupe Hospitalier Sud Reunion, La Reunion, France; 9 Neurological Disorder Center Qatar Biomedical Research Institute, Hamad Bin Khalifa University, Doha, Qatar; 10 Department of medical genetics, Hospices Civils de Lyon, Bron, France; 11 Department of Medical Genetics, Children’s & Women’s Health Centre of British Columbia University of British Columbia, C234-4500 Oak Street, Vancouver, British Columbia V6H 3N1 Canada; 12 Biochemistry and Molecular Genetics Service, Hospital Clínic, Institut d’Investigacions Biomèdiques August Pi I Sunyer (IDIBAPS), Center for Biomedical Network Research on Rare Diseases (CIBERER), Barcelona, Spain; 13 Department of Human Genetics, Graduate School of Medicine, Yokohama City University, Fukuura 3-9, Kanazawa-ku, Yokohama, 236-0004 Japan; 14 Department of Genetics, Microbiology and Statistics, Faculty of Biology, University of Barcelona, av diagonal 643, 08028 Barcelona, Spain; 15 Department of Biochemistry, Hamamatsu University School of Medicine, 1-20-1 Handayama, Higashi-ku, Hamamatsu, 431-3192 Japan; 16 MassGeneral Hospital, Division of Medical Genetics and Metabolism, 175 Cambridge St, Suite 500, Boston, Massachusetts 02114 USA

## Abstract

SETD1B is a component of a histone methyltransferase complex that specifically methylates Lys-4 of histone H3 (H3K4) and is responsible for the epigenetic control of chromatin structure and gene expression. De novo microdeletions encompassing this gene as well as de novo missense mutations were previously linked to syndromic intellectual disability (ID). Here, we identify a specific hypermethylation signature associated with loss of function mutations in the *SETD1B* gene which may be used as an epigenetic marker supporting the diagnosis of syndromic *SETD1B*-related diseases. We demonstrate the clinical utility of this unique epi-signature by reclassifying previously identified *SETD1B* VUS (variant of uncertain significance) in two patients.

## Introduction

Currently, five patients have been described with a microdeletion 12q31.24 and comparable phenotypes [1–5]. The lost fragment of chromosome 12 varied in size and included multiple genes. Labonne et al. [5] identified the smallest overlapping region and proposed two histone modifiers, *KDM2B* and *SETD1B*, as the most probable candidates to be responsible for the microdeletion 12q24.31 syndrome. *SETD1B* encodes a SET domain-containing protein, which is a part of a histone methyltransferase complex. The key role of this complex is methylation of histone 3 on lysine 4 (H3K4), which is enriched in gene promoters and is seen to be highly correlated to gene expression [6]. *KDM2B* is a member of the F-protein family and encodes an enzyme that demethylates H3K36me2/3 and H3K4me3 [7]. Labonne et al. [5] showed that the genetic organization of 12q24.31 is conserved between zebrafish and humans and that *KDM2B* and *SETD1B* were expressed in the brain tissue of both zebrafish and human, suggesting evolutionary conservation of the regulation of these genes [5]. More recently, three patients with de novo point mutations in *SETD1B* have been described [8, 9]. Their phenotypes were similar to patients with a 12q24.31 microdeletion.

Since it has been shown that there is a strong relationship between the methylation of H3K4 and DNA methylation [10–13], we set out to determine whether the *SETD1B* and *KDM2B* aberrations can manifest with a specific DNA methylation signature. For this, a genome wide-methylation analysis was performed on DNA samples from 13 patients with either aberrations of 12q24 (including or not including *KDM2B* and/or *SETD1B* genes) or mutations in *SETD1B* (Table 1). This set of patients included previously described patients and additional cases identified in our laboratory or through GeneMatcher [14].

**Table 1:** Cohort—molecular characteristics

Patient no.	Patient ID	Aberrations	Pathogenicity	Inheritance	SETD1B aberrations/variations	KDM2B aberration	*SETD1B* DNAm signature	Batch	Previously reported
1	1_mut	p.Arg1301*	Pathogenic	de novo	Yes	No	*Yes*	1	No;
2	2_mut	p.Arg1902Cys	Pathogenic	de novo	Yes	No	*Yes*	1	No
3	3_mut	p.Arg1902Cys	Pathogenic	de novo	Yes	No	*Yes*	2	Yes; Hiraide et al. [8]
4	4_mut	p.Arg1885Trp	Pathogenic	de novo	Yes	No	*Yes*	2	Yes; Hiraide et al. [8]
5	5_mut	p.Arg1885Trp	Pathogenic	unknown	Yes	No	*Yes*	2	No
6	6_mut	p.Glu1692del	VUS	unknown	Yes	No	No	1	No
7	7_mut	p.Glu1160Lys	VUS	de novo	Yes	No	No	2	No
8	1_del12q	The minimal deletion:	VUS	Pat. inheritance	No	Yes	No	1	Yes; Chouery et al. [2]
		12q24.3(121150820-122120257)							
		The maximal deletion:							
		12q24.3(121139660-122135589)							
9	2_del12q	The minimal deletion:	Pathogenic	de novo	Yes	Yes	*Yes*	2	No
		12q24.31(121838818-122405204)							
		The maximal deletion:							
		12q24.31(121814901-122423659)							
10	3_del12q	The minimal deletion:	Pathogenic	de novo	Yes	Yes	*Yes*	1	Yes; Labonne et al. [5]
		12q24.31(121895610-122271171)							
		The maximal deletion:							
		12q24.31(121882128-122294222)							
11	4_del12q	The minimal deletion:	Pathogenic	de novo	Yes	No	*Yes*	1	Yes; Qiao et al. [4]
		12q24.31(122255880-123758046)							
		The maximal deletion:							
		12q24.31(122234178-123780094)							
12	5_del12q	The minimal deletion:	VUS	unknown	No	No	No	2	No
		12q24.31q-12q24.32(122844745-127838399)							
		The maximal deletion:							
		12q24.31q-12q24.32(12:122825331-127854607)							
13	dup12q	The minimal duplication:	VUS	Mat. inheritance	No	No	No	1	No
		12q24.12(12:112169989-112313658)							

*Mutations are reported according to NM_001353345.1; Hg19

The minimal deletion/duplication within the given start and end position

The maximal deletion—without the given start and end position (between)

## Results

### Identification of *a SETD1B*-related specific methylation signature

Genomic DNA was obtained from whole blood samples (13 patients and 60 controls), and genome methylation status was analyzed using the Infinium MethylationEPIC BeadChip. The determination of DNAm signature based on HumanMethylation array was previously validated and described in various studies [13, 15–19].

The principal component analysis (PCA) of the data obtained showed two outliers in our cohort: a patient with a microdeletion including *SETD1B* and *KDM2B* (3_del12q; Batch1) and a healthy control (4 days old, batch 2). Estimation of the blood cell types in patient 3_del12q showed an unexpected distribution of cell types (99% of B lymphocytes). Both outliers were excluded from further group analysis. Quality control (QC) of the data, PCA analysis, and estimation of the blood cell type distribution are described in detail in the supplemental information and listed in Additional file 1: Table S1.

Next, a group-based differential methylation analysis was carried out, comparing the DNAm of five patients with pathogenic variants in *SETD1B* to that in controls (*n* = 59). Variants were considered pathogenic if the following was observed: (*i*) variants were de novo and occurred in more than one patient or (*ii*) variants resulted in a premature stop codon. The patients included in the group analysis were patient 1_mut (p.Arg1301*), patients 2_mut and 3_mut (p.Arg1902Cys), and patients 4_mut and 5_mut (p.Arg1885Trp).

**Fig. 1:**
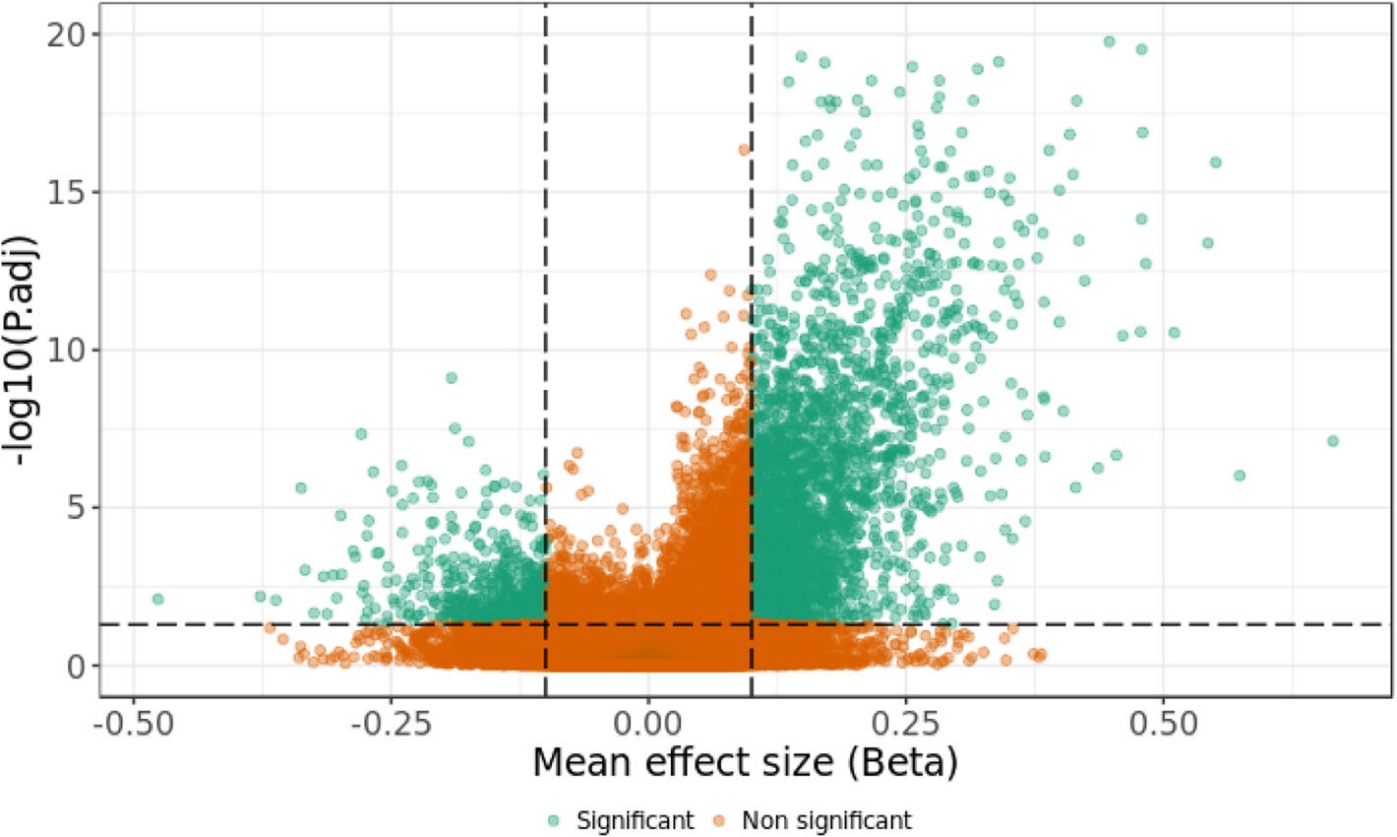
The volcano plot of the methylation difference between patients with certain pathogenic variation in *SETD1B* and healthy individuals (group analysis). The *y*-axis represents a negative log_10_ of adj. *P*-values_M; the *x*-axis represents the different beta values between patients and controls. Each dot on the plot represents a single CpG site. The horizontal, dotted line represents the statistical significance threshold (adj. *P*-values_M = 0.05). The vertical, dotted lines show the effect-size threshold (− 0.1 and 0.1). CpGs with adj. P-value_M lesser than 0.05 and an absolute beta difference higher than 0.1 are highlighted in green

A shift of the genome-wide methylation toward hypermethylation was observed (Fig. 1), which is reflected in the selected significant differentially methylated CpGs (adj. P-value_M < 0.05, absolute beta difference > 0.1). This analysis identified 3340 significant differentially methylated CpGs, out of which more than 82% had a positive beta difference. All significant differentially methylated CpGs identified in this analysis are listed in Additional file 2: Table S2. To further calculate the probability that we would have identified that these 3340 CpGs as significant by chance, we performed an additional permutation analysis on the group labels. 99.6% of 3340 significant differentially methylated CpGs displayed *P* value less than or equal to 0.05. Details of this analysis are described in the additional information and listed in the Additional file 6: Table S6.

**Fig. 2:**
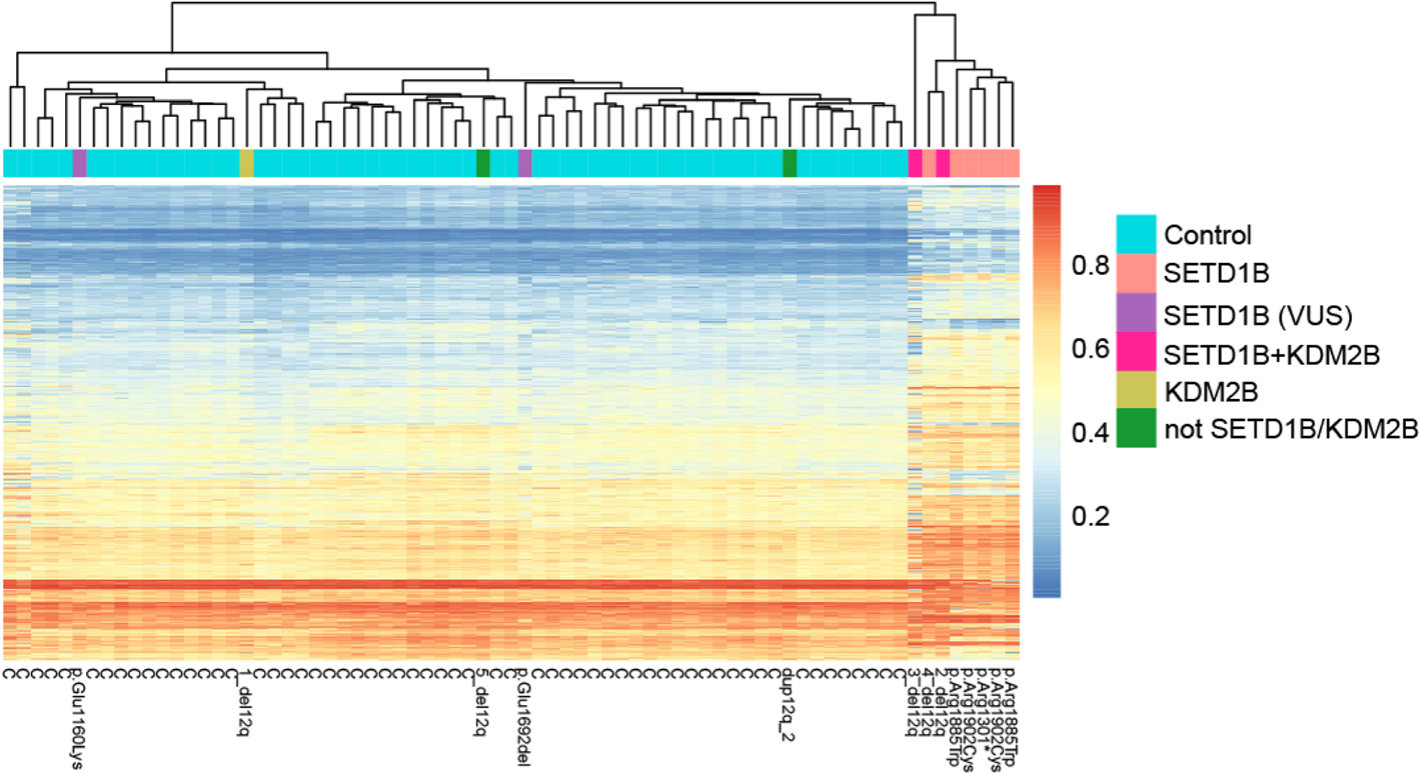
*SETD1B*-related DNAm signature**.** Unsupervised hierarchical clustering of 3340 CpG sites identified in the *SETD1B* group analysis (DNAm of patients with certain pathogenic aberration/variation in *SETD1B* compared to that in healthy controls). C represents controls; aberrations/variations are annotated to patients. Note that the data was obtained from two batches

Next, unsupervised hierarchical clustering of beta values of the identified significant CpG sites (3340 CpGs) for each individual of our cohort was created; 13 patients and 60 controls (Fig. 2). Eight of the 13 patients were clustered in a separate group. All five patients with pathogenic variants in *SETD1B* (patients included in the “*SETD1B*-related” group analysis); two patients with a deletion including *KDM2B* and *SETD1B* (2_del12q, 3_del12q) and one with a deletion including only *SETD1B* (4_del12q) fell into this cluster. Note that although patient 3_del12q had an aberrant blood cell composition, the methylation signature was detectable in this sample. These results demonstrate the robustness of the specific DNAm of the *SETD1B* aberrations/variations. Despite the many variables in the cohort that may have had an impact on the DNAm (different ethnicity, different aberrations/variations, a different method of DNA isolation small sample size, batch, age, and distribution of the cell types), there is a distinct *SETD1B* specific methylation signature. The methylation profile of the patients with a deletion excluding *SETD1B* (1_del12q and 5_del12q_a), a patient that carried a duplication of the 12q region, and two patients with a variant of uncertain significance, in *SETD1B* (6_mut and 7_mut), did not show the *SETD1B-*specific signature.

### Examination of the specificity of the *SETD1B*-related DNAm signature

**Fig. 3:**
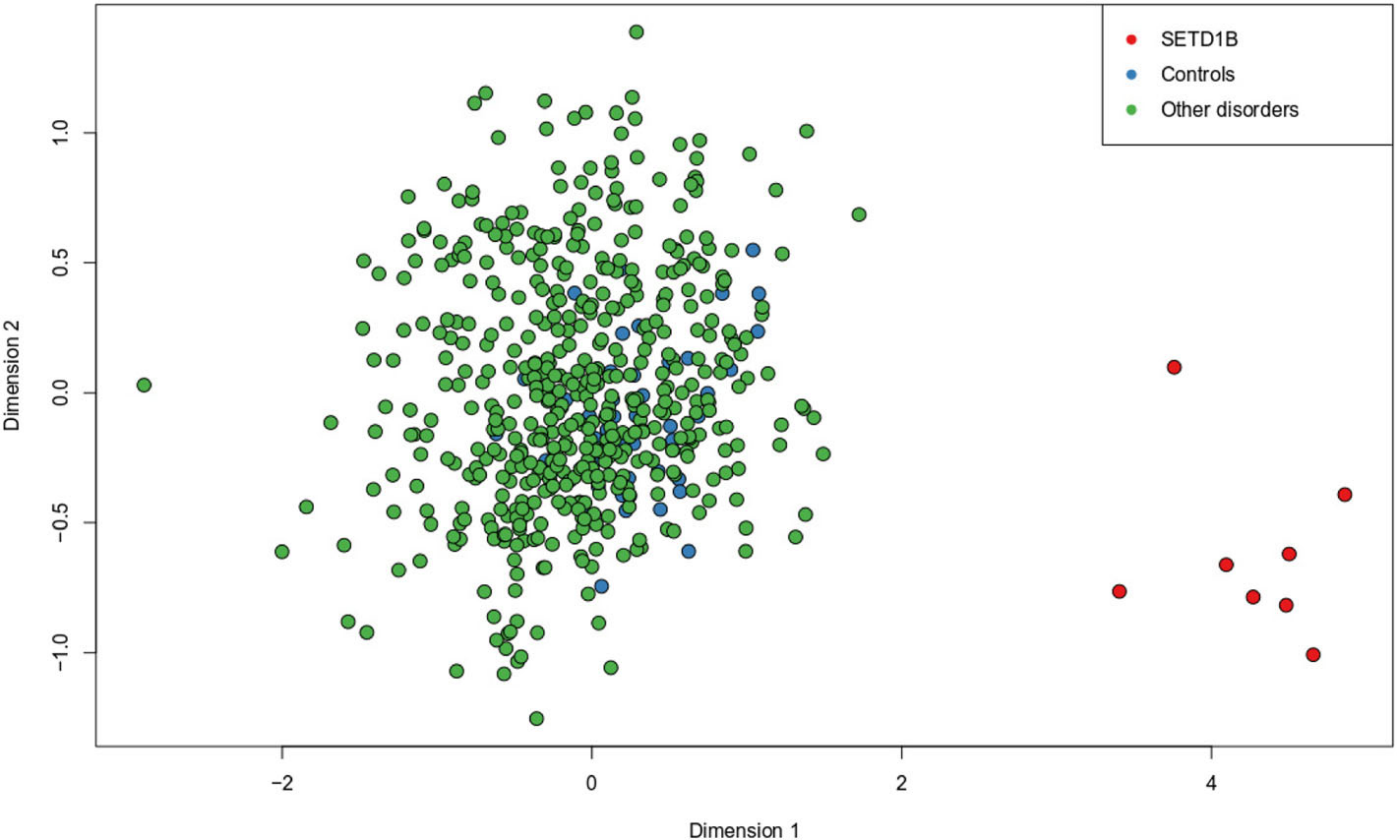
Multidimensional scaling (MDS) of 502 individuals with neurodevelopmental disorders. Red dots represent eight patients with *SETD1B*-related DNAm signature of the current study, blue dots represent controls of the current study, and green dots represent patients with other disorders

We examined whether the DNA methylation signature of *SETD1B*-related syndrome overlaps with that of other neurodevelopmental disorders or syndromes, which in some cases, are caused by mutations in the members of the epigenetic machinery. Using a multidimensional scaling of the methylation values across the CpGs differentially methylated in the *SETD1B*-related syndrome, we examined the methylation profile of a total of 502 individuals with a confirmed diagnosis of various syndromes with previously described epi-signatures including imprinting defect disorders [16, 17, 20] (Angelman syndrome, Prader–Willi syndrome, Silver–Russell syndrome, and Beckwith–Wiedemann syndrome), BAFopathies (Coffin-Siris and Nicolaides-Baraitser syndromes), Autosomal dominant cerebellar ataxia, deafness, and narcolepsy, Floating–Harbor syndrome, Cornelia de Lang syndrome, Claes–Jensen syndrome, ADNP syndrome, ATRX syndrome, Kabuki syndrome, CHARGE syndrome, Fragile X syndrome, trisomy 21, Williams syndrome, and Chr7 duplication syndrome (Fig. 3). All of these patients showed a DNA methylation pattern different from the *SETD1B*-related syndrome and were clustered with controls, indicating that the identified epi-signature is highly specific to *SETD1B* loss of function.

### Identification of the *SETD1B*-related differentially methylated regions

Using the “bumphunte*r”* R-package, four genomic regions differentially methylated between patients with pathogenic variants in *SETD1B* (as defined above) and controls were identified (minimum three differentially methylated CpGs in a region; family-wise error rate (Fwer) < 0.05) (Table 2). All four regions were hypermethylated in patients and located in the regulatory clusters of active promoters, enhancers, and DNAse hypersensitivity (UCSC Genome Browser on Human; GRCh37/hg19 [21]), three of which were annotated to genes *(i) KLHL28, FAM179B; (ii) RUNX1;* and *(iii) BRD2.*

**Table 2:** DMRs identified in the group analysis of certain pathogenic aberrations/variants in SETD1B

Chr	Start	End	Value	L	ClusterL	Fwer	Gene_Name
chr6	26195488	26195995	0,45	5	5	0,002	
chr14	45431885	45432516	0,40	4	21	0,014	*KLHL28;FAM179B*
chr21	36258423	36259797	0,21	13	13	0,02	*RUNX1*
chr6	32942063	32943025	0,26	11	128	0,026	*BRD2*

Value –represents the difference between patient end controls

*L*– number of differentially methylated CpGs in the detected region, *Cluster L*– number of CpGs in the genomic cluster, *Fwer*– family-wise error rate

### Analysis of the genomic distribution of the CpG sites in the *SETD1B* DNAm signature

**Fig. 4:**
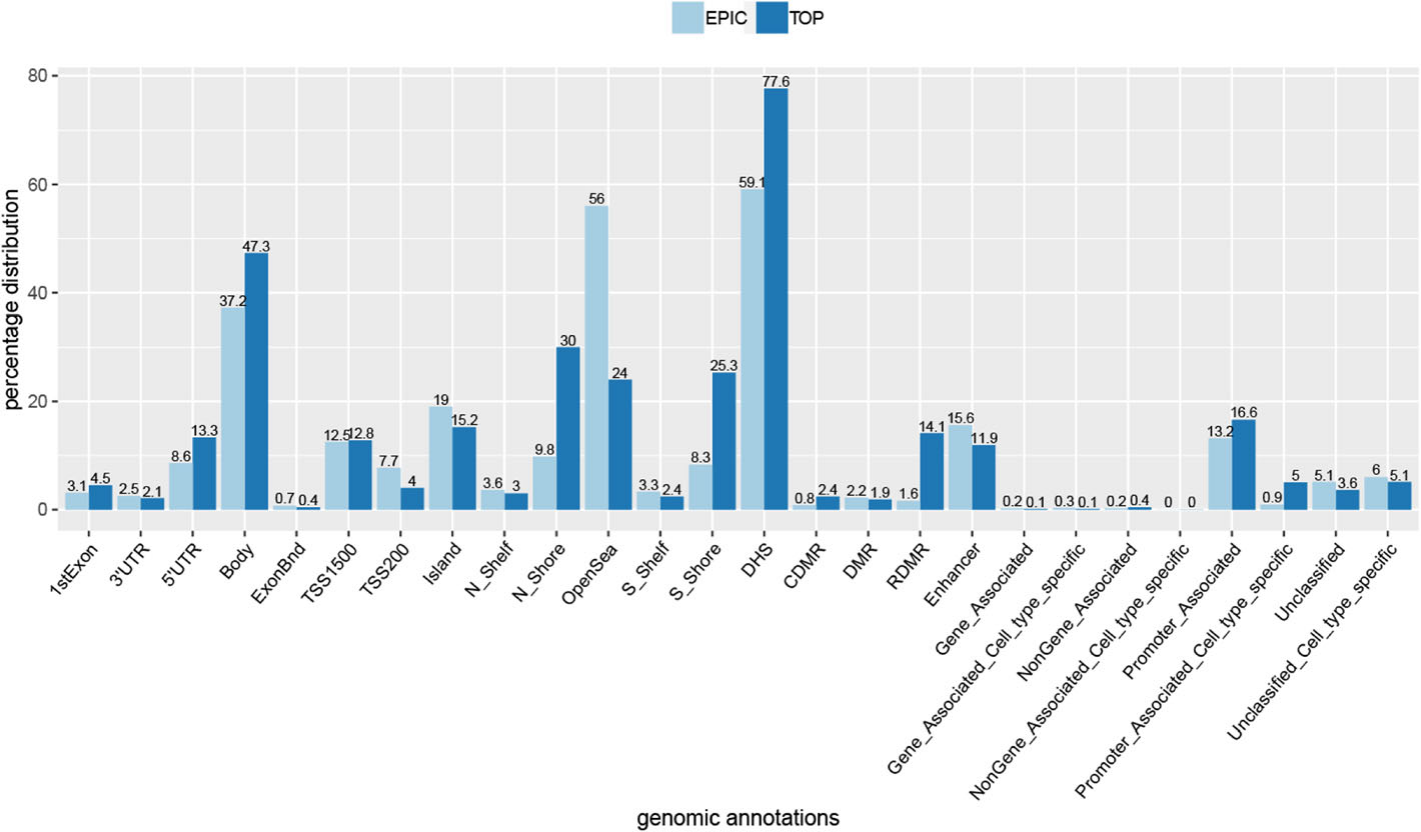
Genomic distribution of the significant differentially methylated CpG sites identified in group analysis according to the genomic annotations of the epic array. The light blue bars (EPIC) represent all the informative probes included in the data (777,148 CpGs) and the dark blue bars the CpGs identified in the group analysis (TOP; 3340 CpGs). The numbers on the top of the bars represent the percentage distribution of CpGs for each category. All categories are listed in the supplemental information—Infinium Methylation EPIC Manifest Column Headings®. This comparison demonstrates the enrichment in the body (between the ATG and stop codon), DHS–DNase I hypersensitivity site, RDMR–reprogramming-specific differentially methylated region, promoter-associated, and promoter-associated cell-type specific

An analysis of the genomic distribution of the CpG sites identified in the group analysis was conducted. This showed an over-representation of CpGs in the gene body, DNase hypersensitivity sites (DHS), CpG island S-shore, reprogramming differentially methylated regions (RDMR), and in promoter-associated sites (Fig. 4). These results demonstrate that the disrupted methylation related to the *SETD1B* function is enriched in the regulatory parts of the genome.

### Over-representation analysis (ORA) of CPGs in the *SETD1B* DNAm signature

To identify the processes involved in the development of the phenotype, ORA analysis based on gene names associated with the 3340 identified significant methylated CpGs using WEB-based GEne SeT AnaLysis Toolkit [22] was performed. The analysis for biological processes displayed enrichment for genes with a function in chromosome organization, regulation of organelle organization, cell cycle, and regulation of cell death. ORA for molecular function demonstrates enrichment for genes with a role in the regulation of gene activity, such as RNA binding, protein domain-specific binding, regulatory region nucleic acid binding, and transcription regulatory region DNA binding. ORA for the human phenotype (top 10 highest ranked features) showed enrichment in genes related to facial and posture abnormalities. The results of ORA are summarized in Table 3. Note that ORA analysis is very general and the results should be interpreted with caution.

**Table 3:** Summary of the ORA

Gene ontology: biological processes							
	Description	*C*	*O*	*E*	*R*	pValue	FDR
GO:0051276	Chromosome organization	1143	165	97.67	1.69	5.59E−12	5.08E−08
GO:0033043	Regulation of organelle organization	1245	175	106.39	1.64	1.16E−11	5.26E−08
GO:0007049	Cell cycle	1739	223	148.60	1.50	1.15E−10	3.48E−07
GO:0006915	Apoptotic process	1911	239	163.30	1.46	2.52E−10	5.74E−07
GO:0010941	Regulation of cell death	1648	210	140.83	1.49	7.83E−10	1.42E−06
GO:0006325	Chromatin organization	741	112	63.32	1.77	1.35E−09	1.90E−06
GO:0033554	Cellular response to stress	1867	231	159.54	1.45	1.47E−09	1.90E−06
GO:0010942	Positive regulation of cell death	660	102	56.40	1.81	2.28E−09	2.60E−06
GO:0010629	Negative regulation of gene expression	1733	216	148.09	1.46	3.00E−09	2.87E−06
GO:0034613	Cellular protein localization	1815	224	155.10	1.44	3.44E−09	2.87E−06
Gene ontology: molecular function							
GO:0003723	RNA binding	1603	203	131.47	1.54	7.52E−11	8.28E−08
GO:0019904	Protein domain specific binding	684	106	56.10	1.89	8.82E−11	8.28E−08
GO:0001067	Regulatory region nucleic acid binding	898	129	73.65	1.75	1.39E−10	8.70E−08
GO:0044212	Transcription regulatory region DNA binding	896	128	73.48	1.74	2.38E−10	1.12E−07
GO:0043565	Sequence-specific DNA binding	1097	146	89.97	1.62	1.87E−09	7.02E−07
GO:0003690	Double-stranded DNA binding	915	126	75.04	1.68	3.41E−09	1.07E−06
GO:0000976	Transcription regulatory region sequence-specific DNA binding	781	111	64.05	1.73	5.41E−09	1.45E−06
GO:1990837	Sequence-specific double-stranded DNA binding	823	115	67.50	1.70	7.55E−09	1.77E−06
GO:0000977	RNA polymerase II regulatory region sequence-specific DNA binding	729	103	59.79	1.72	2.69E−08	5.62E−06
GO:0001012	RNA polymerase II regulatory region DNA binding	735	103	60.28	1.71	4.11E−08	7.72E−06
Human Phenotype Ontology							
HP:0002346	Head tremor	20	10	1.87	5.36	3.48E−06	0.016253
HP:0011337	Abnormality of mouth size	269	43	25.09	1.71	2.13E−04	0.167774
HP:0004097	Deviation of finger	320	49	29.85	1.64	2.23E−04	0.167774
HP:0000311	Round face	73	17	6.81	2.50	2.80E−04	0.167774
HP:0000219	Thin upper lip vermilion	137	26	12.78	2.03	2.84E−04	0.167774
HP:0011228	Horizontal eyebrow	8	5	0.75	6.70	3.04E−04	0.167774
HP:0005306	Capillary hemangioma	26	9	2.43	3.71	3.59E−04	0.167774
HP:0001894	Thrombocytosis	21	8	1.96	4.08	3.63E−04	0.167774
HP:0100559	Lower limb asymmetry	21	8	1.96	4.08	3.63E−04	0.167774
HP:0000107	Renal cyst	203	34	18.93	1.80	4.21E−04	0.167774

*C* reference genes in the category, *O* observed number of genes in the category, *E* expected number of genes in the category, *R* ratio of enrichment, *pValue p* value from hypergeometric test, *FDR* false discovery rate

### Analysis of a *KDM2B*-related specific methylation signature

**Fig. 5:**
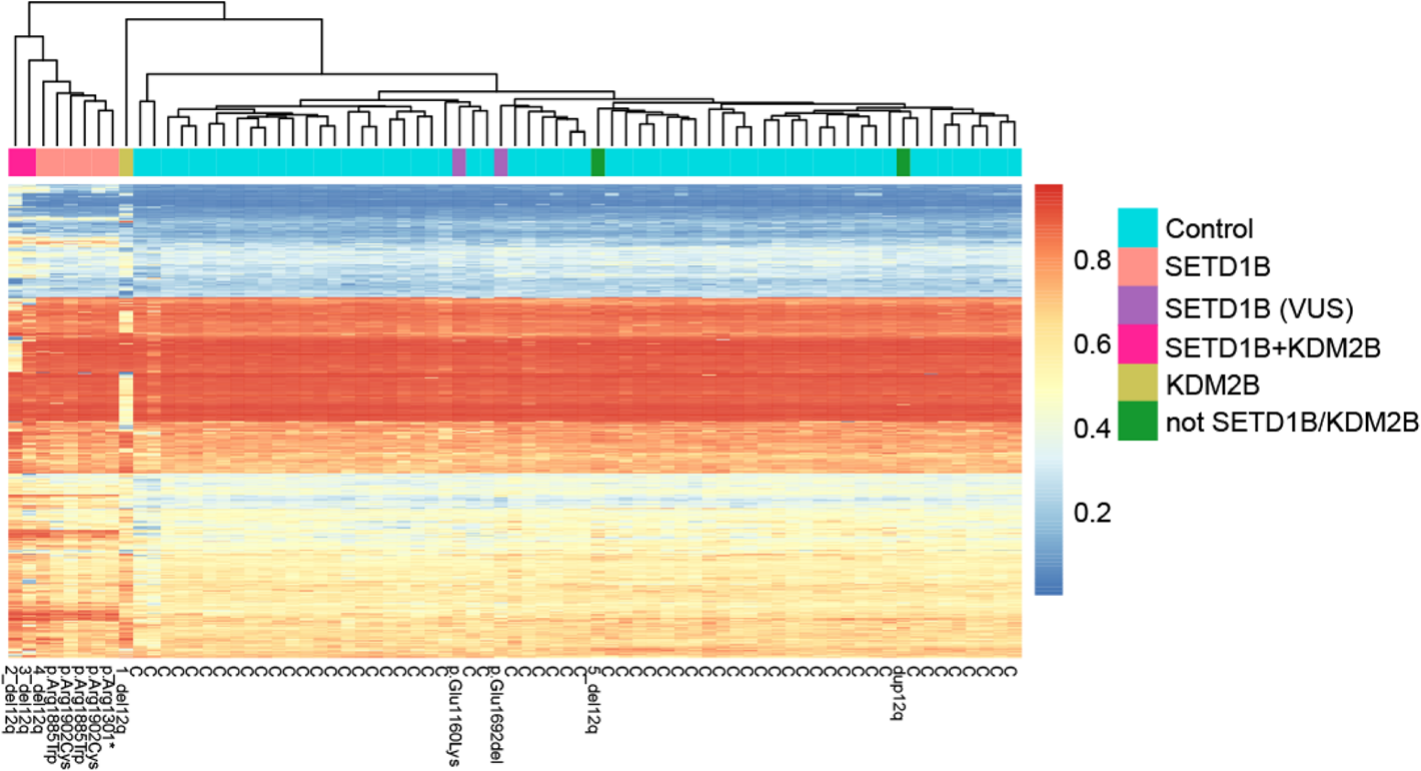
Unsuperviesed hierarchical clustering of the 697 CpG sites identified in KDM2B group analysis. C–represents controls, aberrations/variations annotated to patients. The data was obtained from two batches

Only three patients in this cohort had a deletion of *KDM2B* (1_del12q, 2_del12q, 3_del12q), one of whom presented with a deletion excluding *SETD1B* (1_del12q). Furthermore, of these, patient 3_del12q was excluded from the group analysis due to the heavily disturbed blood cell-type distribution. Despite these limitations, an attempt was made to identify a *KDM2B*-specific signature, running the group analysis of only two patients (1_del12q, 2_del12q) compared to 59 controls. This identified 697 significant differently methylated CpG sites (adj. *P*-value_M < 0.05 and absolute beta difference > 0.1). Nevertheless, the unsupervised hierarchical clustering (Fig. 5) of the 697 identified CpGs did not show any specific methylation signature related to *KDM2B.* The two patients (1_del12q and 2_del12q) were clustered separately from each other, other patients, and healthy controls. Moreover, the *SETD1B*-related specific signature was still strongly marked. All significant differentially methylated CpGs identified in this analysis are listed in Additional file 3: Table S3.

### Identification of the *KDM2B*-related differentially methylated regions

The DMR analysis did not show any significant DMR (minimum of three differentially methylated CpGs in a region; Fwer < 0.05).

### Clinical features

All patients with a *SETD1B* signature-positive methylation profile presented with an intellectual disability. Common features included language delay, epilepsy, and behavioral problems such as autism spectrum disorder and anxiety. Dysmorphisms included full cheeks, full lower lip, macroglossia, and tapering fingers. Delay in motor development was primarily present in patients with a deletion and absent in patients with a point mutation in *SETD1B* (Table 4).

**Table 4:** Summary of clinical features of patients with variation/aberration within the *SETD1B* gene

	Specific SETD1B-related DNAm signature (this study)								Other previously reported patients (not included in this study)		Non-SETD1B DNAm signature	
Clinical features	mut_1 Male 13 years Disrupted SETD1B	mut_2 Male 16 years Disrupted SETD1B	3_mut Male 34 years Disrupted SETD1B	4_mut Female 12 years Disrupted SETD1B	5_mut Male 7 years Disrupted SETD1B	2_del12q Female 12 years Disrupted SETD1B and KDM2B	3_del12q Male 3 years Disrupted SETD1B and KDM2B	4_del12q Female 16 years Disrupted SETD1B	Baple et al. [1] Female 11 years Disrupted SETD1B and KDM2B	Palumbo et al. [3] Female 11 years Disrupted SETD1B and KDM2B	6_mut Male 8 years VUS SETD1B	7_mut Male 10 years VUS SETD1B
Growth parameters at birth												
Height	48 cm	NA	NA	–	47 cm (5th centile)	52 cm (+ 0.45 SD)	NA	NA	NA	NA	NA	NA
Weight	2.9 kg	2.52 kg (2.5 SD)	3.6 kg (+ 1.5 SD)	3.55 kg (+ 1.4 SD) 34.5 cm (+ 1.1 SD)	2.8 kg (9th centile)	3.78 kg (+ 1.45 SD)	NA	NA	4094 g (90–95th centile)	2650 g (5–10th centile)	3260.2 (25–50th centile)	NA
Head circumference	33 cm	NA	NA	NA	33 cm (10th centile)	35 cm (− 0.1 SD)	NA	NA	NA	NA	NA	NA
Growth parameters at last evaluation												
Height	167.5 cm (at 30 years)	193 cm (+ 1.45SD)	NA	NA	1.35 m (+ 1.8 SD)	170 cm (+ 1.2SD) (at 13)	NA	(98th centile)	157.5 cm (98th centile)	(10–25th centile)	13 cm (67th centile)	NA
Weight	111.8 kg (at 30 years)	67 kg (− 0.15SD)	NA	NA	46 kg (>> + 3 SD)	84.9 kg; + 2.5 SD (according to height)	NA	(98th centile)	91.5 kg (98–99,6th centile)	(10–25th centile)	46.8 kg (95th centile)	
Head circumference	60 cm (at 30 years)	NA	NA	NA	51 cm (− 1.2 SD)	48 cm; − 0.97 SD at 3 years	NA	(98th centile)	54.8 cm (75th centile)	(10–25th centile)	54 cm	NA
Dysmorphisms												
Head	–	NA	NA	Normal	NA	Prominent forehead	Narrow face, prominent forehead, plagiocephaly	NA	NA	NA	Very fair hair	NA
Eye	–	Up slant palpebral fissures, proptosis	Thick eye brows	Normal	Thick eyebrows, hypertelorism, sunken eyes, short palpebral fissures	Telecanthu, epicanthus	Hypertelorism	Up slanting palpebral fissures, synophrys	NA	NA	Very Fair (blue)	Upslant palpebral fissures, myopia
Ear	–	Normal	Normal	Normal	Thick helix	Tags preauriculair	Folded ear ridges	Small, low set and posteriorly rotated	Large, narrow with thick helix and rotated	Large and narrow with a thick helix	NA	NA
Nose	–	Asymmetric due to cleft lip	Normal	Normal	Normal	Short upturned nose, large nose bridge	NA	Height nasal bridge, square tip	Broad nasal based	Broad base; high root	NA	NA
Cheeks	Full	Normal	Full	Normal	Full	Full	NA	NA	Full	Full	NA	NA
Lip	–	Cleft lip	Full lower lip	Normal	Full	Full lower lip; short philtrum	NA	NA	Full and everted lower lip	Full and everted lower lip	NA	Malformation of upper lip, prominent upper lip
Mouth	–	Cleft jaw bilateral	NA	Normal	NA	Macroglossia; prognathic	NA	Minor micrognathia	Macroglossia	Macroglossia	NA	NA
Palate	–	Cleft palate	NA	Normal	NA	NA	NA	Narrow palate	High arch	High arch	NA	NA
Teeth	–	Misaligned due to cleft jaw	NA	Normal	Oligodontia	Irregular, oligodontia	NA	Prominent front incisors	Overcrowded	Overcrowded	NA	Malaligned teeth with increased spacing
Developmental delay												
Intellectual disability	Mild – moderate	Mild	Profound	Mild	Profound	+		Moderate	Moderate	moderate to severe	mild-to-moderate	+
Motor development	Walk without support	–	Walk without support	Walk without support	Walk without support	Normally but her movements are not fluent	+	Global developmental delay	Walk with a broad-based gait	global developmental delay	global developmental delay	+
Language delay	+	+	+	+	+	+	+	–	+; few words at 2 years old	–	first words at 3 years	NA
Anxiety	–	–	+	–			+	–	+	+	–	NA
Autism/autistic behavior	–	–	+	+	+	+	+	–	+; at 4 years	+	–	+
Epilepsy/seizures/spasms												
Type	Frontal-temporal	In early childhood absences, alter tonic-clonic seizures	Myoclonic seizures (3y11m)	Myoclonic seizures (2y9m),	NA	NA	Myoclonic seizures	NA	–	Tonic-clonic seizures	No seizures	Tonic-clonic seizures remotely in childhood and more recently complex partial seizures
Fingers abnormality	NA	Fetal pads	Tapering fingers- mild	–	Tapering fingers-mild	Clinodactyly	Tapering fingers	Tapering fingers with prominent fingertip pads	Tapering finger – mild left 4th finger proximally implanted	Tapering fingers – mild	–	Long fingers, widened tips, 5th finger clinodactyly
Toes	Foot pronation	Normal	NA	NA	NA	NA	NA	Bilateral hypo-plastic nails on both halluces	Short toes	NA	–	NA
Hypoglycemia	–	–	–	–	NA	–	NA	NA	+	+	–	NA
Hypotonia	+	–	–	–	NA	–	+	–	+	–	–	NA
Additional findings	Obsessive interest for electronic objects and their accumulation, acute pancreatitis, cholecystectomy, liver steatosis				Urinary continence problems	Umbilical hernia at birth, hyperactivity - PDD NOS/ADHD; obstipation		T cell skin lymphoma on the lower back; hypo-plastic nails, patchy eczema, thick ichthyic skin	Cafe-au-lait spot:1 truncal; large hands and feet; urinary continence problems inverted nipples;		His skin is also very fair	Cerebral visual impairment; ptosis

*NA* not available, “+” feature present, “-” feature absent

## Discussion

Pathogenic changes within the *SETD1B* gene were found to have an associated specific DNAm signature. This specific DNAm was not substantially affected by differences in blood cell distribution and other variables such as technical differences and chromosomal aberrations. The specificity of the DNAm signature was highlighted by the lack of signature in patients carrying a deletion that did not include *SETD1B* or in a patient carrying a duplication of the region or patients with other neurodevelopmental disorders or syndromes. Moreover, we were able to assess the pathogenicity of two variants of unknown clinical significance: p.(Glu1692del) and p.(Glu1160Lys) in patients 6_mut and 7_mut, respectively.

The inheritance of variant p.(Glu1692del) in patient 6_mut was unknown. This variant results in the loss of residue Glu1692. The p.(Glu1160Lys) variant in the 7_mut patient occurred de novo*.* It is a missense variant present at very low frequencies in the general population (5/187386 alleles in the GnomAD database [23]; MAF < 0.01; rs959370052) and affects a weakly conserved amino acid. The methylation profile of both patients did not display a specific *SETD1B* signature, suggesting both variants do not result in a loss of *SETD1B* function and are probably not pathogenic. While patients 6_mut and 7_mut display clinical features compatible with the phenotype caused by *SETD1B* mutations, this is not related to the specific *SETD1B* methylation pattern, indicating that they do not have a *SETD1B*-related disorder.

We detected the specific *SETD1B*-related DNAm signature based on the methylation status of three different pathogenic variants in five patients. An increased sample size would lead to the possibility of detecting differences in DNAm between variants.

Four hypermethylated DMRs were found to be associated with *SETD1B.* The region located on chromosome 6 (chr6: 26195488-26195995; hg19) was not assigned to any gene and was found to be characterized by high DNase hypersensitivity with promoter activity and located in *Homo sapiens* histone cluster 1. Histone 1 (H1) is responsible for chromatin condensation and DNA fragmentation during apoptosis [24, 25]. Note that the apoptotic process, regulation of cell death, and chromatin condensation were enriched in ORA (biological processes) of CpG sites of the *SETD1B*-related DNAm-specific signature. Another hypermethylated region on chromosome 6 (chr6: 32942063-32943025; hg19) was assigned to the *BRD2* gene. It displays promoter and enhancer activity and overlaps exon 3 of *BRD2.* Pathak et al. [26] reported hypermethylation in another locus (CPG75) near the promoter of *BRD2* as implicated in juvenile myoclonic epilepsy (JME) [26]. Hypermethylation of this locus was found to be associated with a single nucleotide polymorphism (rs3918149). Schultz et al. [27] could not confirm this association in the German population. However, in 2007, Cavalleri et al. published the results of genotyping rs3918149 variant across five independent JME cohorts, observing a significant effect of this SNP on epilepsy in the British and the Irish cohorts, but not in those of the German, Australian, and Indian [28]. Although the association of *BRD2* and epilepsy is not clear, we tentatively speculate that the hypermethylation detected in *BRD2* in our cohort may play a role in the occurrence of epilepsy in these patients. Two other hypermethylated DMRs detected in the *SETD1B*-related group analysis were found to be located on chromosomes 14 and 21 (chr14:45431885-45432516; chr21:36258423-36259797; hg19) and assigned to genes *KLHL28*, *FAM179B*, and *RUNX1*. The former covers a CpG island with promoter activity and a DNAse hypersensitivity cluster (exon 1 of *FAM179B*) while the latter corresponds to a CpG island with promoter activity at exon 4 of *RUNX1* and a DNAse hypersensitivity cluster. The biological function of these genes could not be related to clinical features in our cohort; however, their localization in genomic regulatory regions suggests a role in *SETD1B-*related disorders.

A comparison of phenotypes of patients with a *SETD1B* DNAm signature showed overlapping clinical features such as intellectual disability, language delay, autism, seizures, full cheeks, and tapering fingers (Table 4). Interestingly, two patients presenting with the microdeletion, involving also *KDM2B*, were initially diagnosed with Beckwith Wiedemann syndrome (BWS) because of overgrowth and macroglossia, which are typical for BWS (MIM 130650). Hiraide et al. (2018) suggested that the deletion of *KDM2B* could be a possible reason for an overgrowth phenotype in these two patients [8]. Moreover, a *KDM2B* missense mutation (c.2503G > A) was identified to be associated with “paunch calf syndrome” [29]. The characteristic features of this syndrome include abdominal distension and tongue protrusion that are comparable with abdominal wall defects and macroglossia, features that are characteristics for BWS [30].

The results of this study show a strong effect of *SETD1B* function on DNA methylation*. SETD1B* is a known histone modifier that produces trimethylated histone H3 at Lys4 (H3K4me3), which may play a role in blocking of the de novo DNA methylation in some genomic regions. DNMT3L ((cytosine-5)-methyltransferase 3-like), which stimulates de novo DNA methylation, interacts only with unmodified H3K4. The methylation of H3K4 disables this interaction [31]. The loss of the function of *SETD1B* may lead to the insufficient production of H3K4me3 and, thereby, hypermethylation of the DNA in specific loci. Indeed, 82% of differentially methylated CpGs in patients with a *SETD1B* pathogenic variant were hypermethylated. The 18% of differentially methylated CpGs that were hypomethylated remain unexplained by this mechanism, but these may be secondary effects, caused by altered expression of target genes of *SETD1B.*

Syndromic disorders have often similar clinical features. Genetic testing has multiple limitations. For instance, the resolution often prevents it from detecting low-frequency mosaicism. Moreover, the reason underlying the clinical features can occasionally not easily be inferred from the variants if variant occurs in non-coding regions, contiguous genes are deleted, or if they have been annotated as VUS. Examination of specific DNAm signatures was previously described as a powerful solution in the classification of various unresolved cases including syndromic Mendelian disorders, imprinting disorders, repeat expansion disorders, and uncertain clinical diagnosis with VUS [16, 17] and has therefore been proposed as a novel molecular diagnostic test. Our results reinforce this observation indicating that the specific DNAm signature has a diagnostic value and can be used as an additional diagnostic test to resolve variants of unknown significance in *SETD1B*.

Due to the small sample, we were unable to determine whether the loss of the *KDM2B* caused a specific DNAm signature. Studies including a sufficient number of patients are needed to solve this. The other limitation of our study was the technical differences between samples. Different DNA isolation methods between samples may influence the results.

## Methods

### Patients

**Fig. 6:**
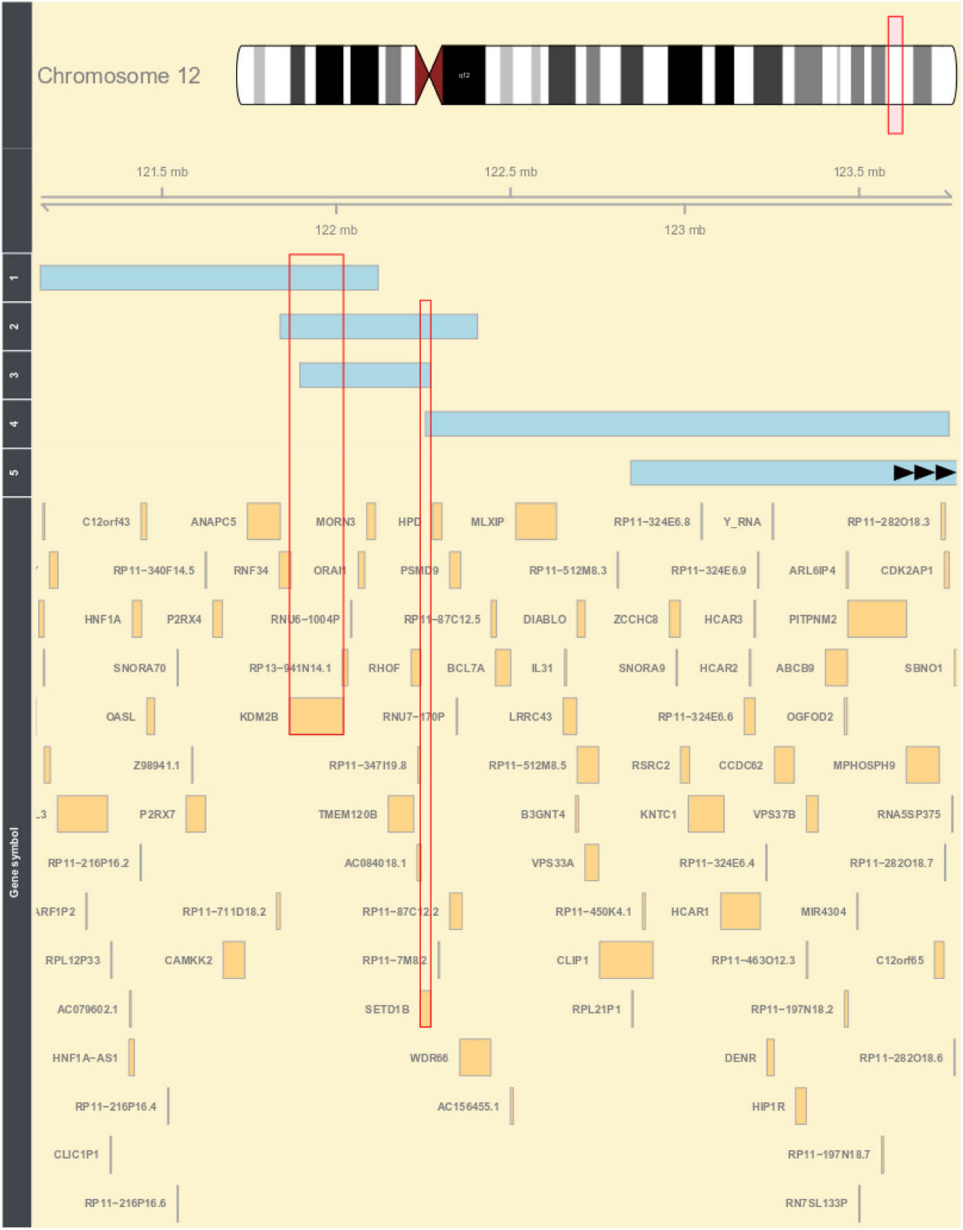
Comparison between deleted regions in patients with a microdeletion of 12q24.31. The light blue bars represent the deleted regions for individual patients. Numbers 1, 2, 3, 4, and 5 represent patients 1_del12q24.31, 2_del12q24.31, 3_del12q24.31, 4_del12q24.31, and 5_del12q24.31, respectively. The red frames highlight genes *SETD1B* and *KDM2B*. Note: microdeletion of patient 5_del12q24.31 has not been fully displayed on the plot and does not overlap *KDM2B* and *SETD1B*

Whole blood DNA samples from 13 individuals were collected for the methylation study. Seven patients had point mutations in *SETD1B*, which were identified by whole-exome sequencing (WES), and five chromosomal 12q24.12-32 aberrations. One of the five patients had the deletion involving *KDM2B* (1_del12q), two the deletion of both *KDM2B* and *SETD1B* (2-del12q, 3_del12q), one the deletion on *SETD1B* (4_del12q), one the deletion not involving *KDM2B* and *SETD1B*, and one the duplication of 12q24.12 not involving *KDM2B* and *SETD1B*. Table 1 shows the genetic aberrations and inheritance of the patients included in the analysis. Figure 6 depicts the comparison between the deleted regions and genes in patients with microdeletions of 12q24.31 from the cohort (according to Hg19). Informed consent was obtained for each patient.

### Healthy controls

Whole blood DNA samples were collected from 60 healthy individuals.

Cohort details are listed in Additional file 4: Table S4.

### Methylation EPIC array

The samples were divided into two batches: the first contained seven DNA samples from the patients (two females and five males) and 40 samples from the healthy controls (20 females and 20 males) and the second contained six DNA samples from the patients (two females and four males) and 20 from the healthy controls (ten females and ten males). The samples were randomized and sent to GenomeScan in Leiden (ISO/IEC 17025 approved), where the bisulfite treatment and the hybridization to the Infinium Methylation EPIC array (Illumina) were processed. The raw methylation data were obtained and the quality (QC) of the data assessed using the MethylAid script in R. (GenomeScan’s Guidelines for Successful Methylation Experiments Using the Illumina Infinium® HumanMethylation BeadChip).

### Normalization and data analysis

The EPIC array data was loaded onto the R software and normalized using the *preprocessFunnorm* function of “minfi” R package [32]. All probes containing SNPs (MAF > 0.01), cross-hybridization probes, and probes located on the sex chromosomes were excluded; 776,920 probes remained for analysis. The beta values (ratio of the methylated probe intensity ranging from 0 to 1) were obtained for all the patients from the cohort. Row beta values were normalized and PCA carried out.

### Estimation of the blood cell type distribution

White blood cell type was estimated for each patient using *estimateCellCounts* function in R “FlowSorted.Blood.EPIC” package [33]. The counts were calculated for CD8T (cytotoxische T cell), CD4T(T helper cells), NK (natural killer cells), B cell (B lymphocytes), mono (monocytes), and gran (granulocytes). The *P* value was calculated for each patient of our cohort (13 patients), for each cell type (Crawford-Howell *t* test; R software). Subsequently, the Bonferroni correction was applied for 78 tests (six cell types × 13 patients). We assume that the distribution of the cell types was significantly disturbed if the Bonferroni-corrected *P* value for the cell types was less than 0.05.

### Group analysis and identification of CpG sites for the DNAm-specific signature

DNA methylation of patients in the groups (five patients in the *SETD1B*-related group and two in the *KDM2B*-related group) were compared with methylation in a group of 59 healthy controls using the “minfi” R-package. The design model was corrected for age, gender, batch, and cell distribution. The beta values were obtained and logit transformed into *M* values. The adjusted *P* values for the *M* values were calculated, and the significance threshold was 0.05. Finally, to avoid false-positive results, CpG sites with an effect size of at least 10% difference in an average of DNAm between patient groups and the control group were selected. In this way, we identified 3340 and 697 differentially methylated CpGs in the *SETD1B*-related group and *KDM2B*-related group, respectively.

### Analysis of a specific methylation signature

Beta values of CpGs selected in the group analyses were used to perform the unsupervised hierarchical clustering (“pheatmap” R-package). Two heatmaps were created, one for the *SETD1B*-related group and the other for the *KDM2B*-related group. Each heatmap was created for all individuals in the cohort (13 patients and 60 controls).

### Examination of the specificity of the SETD1B-related DNAm signature

Whole blood DNA samples were collected from 502 patients with various neurodevelopmental syndromes. To compare the methylation values of our cohort with these additional samples, we performed re-normalization, according to the Illumina normalization method, with background correction using the “minfi” R-package. To select significant differentially methylated *SETD1B*-related CpGs, we used similar filtering steps for these in the *SETD1B*-related group analysis namely, a corrected *P* value less than 0.05 and an effect size of at least 10% difference. Correlated probes with *r*^2^ higher than 0.8 were removed from this analysis. Multidimensional scaling (MDS) was used to examine the DNA methylation profiles. All samples used in this analysis and the details of the method were fully described by Aref-Eshghi et al. [16, 17]. The list of 502 samples used in this specific analysis is listed in Additional file 5: Table S5.

### Identification of differentially methylated regions

To identify the DMRs between patient and control groups, a “bumphunter” R-package was used. The design model was corrected for age, gender, batch, and cell distribution.

The *P* value for each region was calculated and multiple testing applied according to the family-wise error rate. The significant DMRs were selected based on the two filter steps: (i) Fwer < 0.05 and (ii) at least three differentially methylated CpGs within the region (L > 2).

### ORA—WEB-based Gene Set Analysis Toolkit

ORA were carried out for the first and unique gene symbol annotated to the CpGs identified during group analysis (according to the Infinium MethylationEPIC v1.0 B4 Manifest File). Basic parameters were as follows: organism–human, method–ORA, functional database–gene ontology (biological process and molecular function), and reference set for enrichment analysis–genome protein-coding. Advanced parameters were as follows: minimum number of genes for a category–5, maximum number of genes for category–2000, multiple test adjustment–Benjamini-Hochberg (BH), significant level–top 10, number of categories expected from set cover–10, number of categories visualized in the report–40, and color in DAG–continuous.

## Supplementary information

**Additional file 1: Table S1.** The estimation of the cell types distribution and the calculation of *P*-values of the cell types distribution.

**Additional file 2: Table S2.** Significant differentially methylated CpGs identified in the *SETD1B*-related group analysis.

**Additional file 3: Table S3**. Significant differentially methylated CpGs identified in the *KDM2B*-related group analysis.

**Additional file 4: Table S4.** Cohort details.

**Additional file 5: Table S5.** The list of 502 samples used in the examination of the specificity of the *SETD1B*-related DNAm signature.

**Additional file 6: Table S6.** Contains adjusted P-values for M values and empirical *p*-values for 3340 significant differentially methylated CpGs calculated in the *SETD1B*-related group analysis and permutation analysis, respectively.

## Data Availability

All HumanMethylation450 data are available on request.

## Abbreviations

BWS: Beckwith Wiedemann syndrome
DHS: DNase I hypersensitivity site
DMR: Differentially methylated region
Fwer: Family-wise error rate
ID: Intellectual disability
JME: Juvenile myoclonic epilepsy
MAF: Minor allele frequency
ORA: Over-representation analysis
PCA: Principal component analysis
QC: Quality control
RDMR: Reprogramming Differentially Methylated Regions
VUS: Variant of uncertain significance
WES: Whole-exome sequencing
